# Ionizing radiation leads to exosome secretion in macrophages through MYC-mediated pathways

**DOI:** 10.1371/journal.pone.0336322

**Published:** 2025-11-05

**Authors:** Hanui Lee, Gyeong Han Jeong, Geun-Joong Kim, Seung Sik Lee, Byung Yeoup Chung, Hyoung-Woo Bai

**Affiliations:** 1 Research Division for Biotechnology, Advanced Radiation Technology Institute (ARTI), Korea Atomic Energy Research Institute (KAERI), Jeongeup, Republic of Korea; 2 Department of Biological Sciences and Research Center of Ecomimetics, College of Natural Science, Chonnam National University, Gwangju, Republic of Korea; 3 Department of Radiation Science, University of Science and Technology (UST), Daejeon, Republic of Korea; Tarbiat Modares University, IRAN, ISLAMIC REPUBLIC OF

## Abstract

Exosomes are cell-derived vesicles that play a crucial role in intracellular communication and are promising biomarkers for therapeutic applications. Despite their significant potential, the application of exosomes as biological therapeutics is limited by their low yield and inconsistent production quality. Ionizing radiation is known to enhance exosome release; however, this effect has been primarily studied in cancer cells. Given the critical role of macrophages in immune regulation and their potential for exosome-based therapies, we investigated the impact of gamma radiation on the secretion of macrophage-derived exosomes. This study demonstrated that gamma radiation significantly enhanced exosome release by both naïve and polarized macrophages. This effect was associated with the overexpression of Myh10 and Myo5b, the motor proteins that play crucial roles in exosome biogenesis and secretion. Furthermore, RNA sequencing and western blot analyses identified the EGFR/IGFR-MYC signaling axis as a key upstream pathway regulating the expression of Myh10 and Myo5b, thereby accelerating exosome secretion. These findings provide a deeper understanding of the molecular mechanisms underlying radiation-induced exosome secretion from macrophages and offer a novel strategy for optimizing exosome production to advance exosome-based therapeutic applications.

## 1. Introduction

Exosomes are small vesicles with diameters ranging from 50 to 200 nm. They are secreted by all cell types and play a crucial role in mediating intercellular communication [1]. Exosomes have been found to contain various biological molecules, including proteins, lipids, and nucleic acids, that originate from the host cells [2]. For instance, exosomes derived from the serum of patients with pancreatitis significantly overexpress the inflammatory protein S100A9 [3]. Similarly, neuron-derived exosomes contain synaptic proteins and asparaginases that participate in synaptic plasticity and long-term memory [4,5]. Because of these characteristics, exosomes isolated from biological fluids exhibit disease-specific molecular signatures, making them promising biomarkers for disease diagnosis. Furthermore, their unique properties highlight their potential as novel agents for biological therapies.

Among the various cell types that secrete exosomes, macrophages are particularly noteworthy because of their central role in regulating immune responses, such as inflammation and tissue regeneration [6]. Their phenotypes are highly diverse and they can be polarized into two states, M1 and M2, through interactions with microenvironmental stimuli. M1 macrophages are typically induced by lipopolysaccharide (LPS) and interferon (IFN)-γ, leading to inflammatory responses, whereas M2 macrophages exhibit anti-inflammatory properties and are involved in tissue regeneration [7,8]. Exosomes derived from M1 macrophages have been shown to repolarize M2 tumor-associated macrophages into the M1 phenotype within tumor sites [9]. Meanwhile, exosomes secreted from M2 macrophages have been reported to induce wound healing [10]. Therefore, there has been considerable interest in utilizing exosomes derived from polarized macrophages as anti-inflammatory or wound-healing agents.

The therapeutic potential of exosomes has been extensively investigated across various therapeutic fields, including immune modulation and drug delivery [11,12]. Despite its considerable potential, much of the research remains in the preclinical stages [13]. This is primarily because of the challenges in maintaining consistency and achieving therapeutic dosage yields [14,15]. Various approaches have been explored to enhance exosome secretion. For example, electrical stimulation can activate Rho signaling and promote vesicle formation [16]. Another strategy involves inhibiting endosomal regulators to prevent the disassembly of multivesicular bodies and increase exosome secretion [17,18]. Furthermore, metabolic stress conditions, such as glucose deprivation or hypoxia, have been shown to activate stress-induced signaling pathways, promoting exosome release [19]. However, despite these advances, these methods still present limitations concerning scalability, reproducibility, and potential impact on exosome cargo composition, which may impact therapeutic applicability. Therefore, innovative engineering strategies are required to regulate exosome release. In this sense, several studies suggest that ionizing radiation may offer valuable insights into the regulation of exosome secretion [20,21].

The ability of ionizing radiation to enhance the release of exosomes from cancer cells has been extensively studied [22]. For instance, Pszczółkowska et al. demonstrated that alpha radiation induces alterations in the exosome release profiles of PC3 and DU145 cells [23]. However, despite the critical role of macrophages in host defense and their promising therapeutic applications, comprehensive studies investigating the effects of ionizing radiation on exosome release from normal immune cells, such as macrophages, are notably limited compared to cancer cells. Furthermore, studies of the mechanism of polarized-macrophage-derived exosome release under ionizing radiation are limited. Understanding the precise mechanisms of exosome release associated with radiation and macrophage subtypes is essential for optimizing exosome-based therapeutics and developing novel strategies for disease treatment. In this study, the impact of ionizing radiation on exosome secretion from macrophages was comprehensively investigated, elucidating the major molecular regulators and signaling pathways involved.

## 2. Results

### 2.1 Isolation and characterization of macrophage-derived exosomes

RAW264.7 macrophages were polarized into M1 and M2 phenotypes using LPS and IFN-γ for M1 polarization, and IL-4 for M2 polarization. Flow cytometry analysis showed that CD86 expression was significantly increased in M1 macrophages, while CD206 expression was significantly elevated in M2 macrophages compared to M0 cells, confirming successful polarization. Additionally, RNA-sequencing (RNA-seq) analysis supported the effectiveness of the polarization by demonstrating that the expression of M1 markers was upregulated in M1 macrophages, while M2 markers were upregulated in M2 macrophages (S1 Fig).

**Fig 1:**
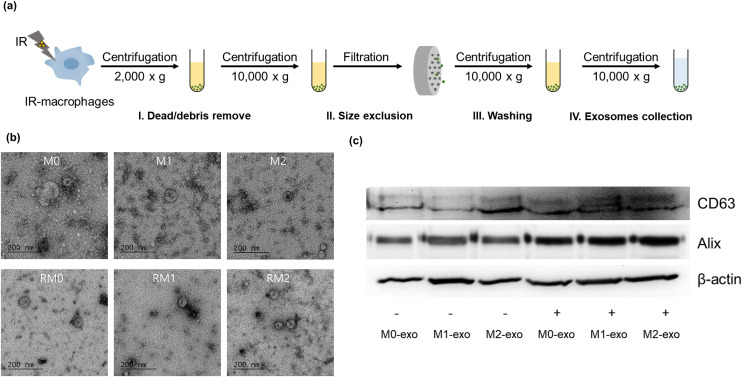
Characterization of exosomes isolated from macrophages. **(a)** Schematic representation of exosome isolation from culture media using an ultracentrifugation method. **(b)** Electron micrograph of exosomes derived from macrophages. **(c)** Expression of CD63, Alix, and β-actin in M0, M1, M2, irradiated **(R)**M0, RM1, and RM2 RAW264.7-cell-derived exosomes. Cells were treated with IFN-ɣ (10 ng/mL) and LPS (0.1 μg/mL) for 24 h to polarize them to the M1 macrophage phenotype, and with IL-4 (10 ng/mL) for 24 h to induce the M2 macrophage phenotype. Protein expression was analyzed by western blotting.

Exosomes derived from irradiated macrophages were isolated using a conventional ultracentrifugation method as outlined in the workflow shown in Fig 1a, and a conventional ultracentrifugation method. The biological properties of the isolated exosomes were then validated. The morphology and structure of the exosomes were analyzed using electron microscopy.

**Fig 2:**
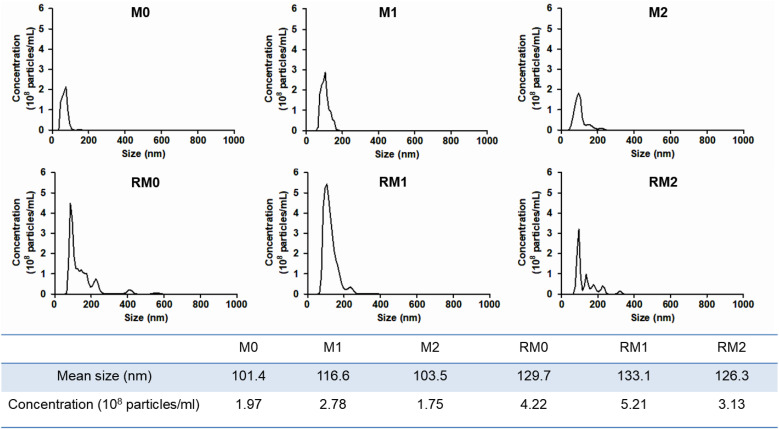
Size distribution and concentration of the exosomes. Exosomes secreted by M0, M1, M2, RM0, RM1, and RM2 macrophages were analyzed using a Nanosight NS300 instrument.

As shown in Fig 1b, electron microscopy analysis confirmed the characteristic round or cup-shaped morphology of isolated exosomes, with the majority exhibiting diameters ranging from 50 to 100 nm. To clearly confirm successful exosome isolation and purity, the expression of both positive and negative exosome-associated protein markers was analyzed by western blotting. Fig 1c clearly shows robust expression of canonical exosome markers (CD63 and ALIX) in all isolated exosome samples. It was observed that the total protein concentration of exosomes, and consequently the signal intensity of exosome markers, was notably higher in samples derived from gamma-irradiated macrophages, consistent with enhanced exosome release. Fig 2 demonstrates the size distribution and particle concentration of exosomes, as measured by nanoparticle tracking analysis (NTA, Nanosight NS300; Malvern Instruments, Malvern, UK), respectively. The hydrodynamic diameters of exosomes from M0, M1, and M2 macrophages were approximately 100 nm. Notably, following gamma irradiation, the sizes of the RM0, RM1, and RM2 exosomes slightly increased to approximately 130 nm. The particle concentration was consistently within the range of 1–5 x 10^8^ particles/mL across all conditions.

### 2.2 Transcriptome profiling of irradiated M0, M1, and M2 macrophages

**Fig 3:**
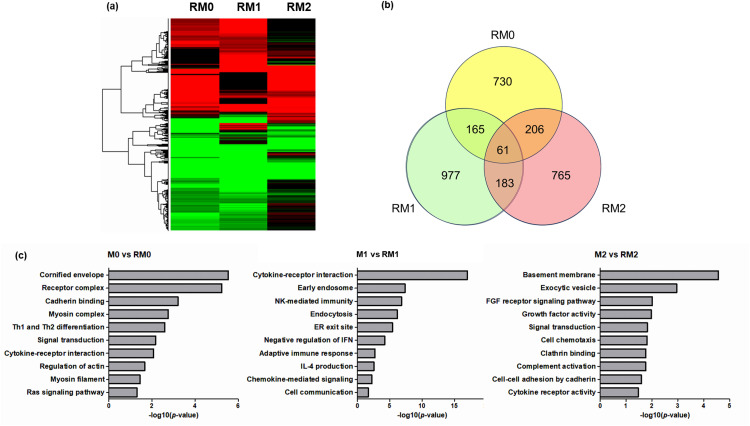
RNA sequencing analysis of gamma-irradiated macrophages. Gamma irradiation induced a comparable gene expression profile across different macrophage subtypes. **(a)** A clustered heat map displays the DEGs in RM0, RM1, and RM2 macrophages compared to their non-irradiated counterparts. **(b)** A Venn diagram illustrates the overlapping and unique DEGs among RM0, RM1, and RM2 groups identified through RNA sequencing. **(c)** Functional annotation of upregulated genes in irradiated macrophages, based on GO enrichment, highlights key biological processes associated with these changes.

To investigate the effects of ionizing radiation on exosome secretion, we performed RNA sequencing (RNA-seq). Sequencing data from non-irradiated and irradiated macrophages were analyzed to investigate the effects of ionizing radiation on exosome secretion. The yields and qualities of the sample libraries are presented in Table 1. A general overview of the sequencing data is presented in Fig 3A.

**Table 1:** Summary of sequencing data.

Sample	Clean reads	Clean bases (bp)	GC content	Q20 (%)	Q30 (%)
M0	24,032,835	4,806,567,000	51.85%	98.48	94.16
M1	24,107,153	4,821,430,600	52.28%	98.59	94.64
M2	24,068,470	4,813,694,000	51.51%	98.50	94.39
RM0	24,152,113	4,830,422,600	52.03%	98.59	94.64
RM1	24,071,527	4,814,305,400	50.99%	98.56	94.52
RM2	24,038,824	4,807,764,800	50.47%	98.47	94.20

Clean reads were paired-end reads of clean data.

Despite the distinct polarization states, gamma irradiation induced broadly similar transcriptional responses in M1 and M2 macrophages. As illustrated by the Venn diagram (Fig 3B), we identified 61 consistently upregulated differentially expressed genes (DEGs) common to all three irradiated macrophage subtypes (M0, M1, and M2) relative to their respective control macrophages. To further investigate the underlying functions of these DEGs, gene ontology (GO) enrichment analysis was conducted. Notably, GO functional analysis revealed that biological processes highly relevant to this study, such as “membrane complexes,” “exosome biogenesis,” and “cytokine-mediated signaling pathways,” were among the top 10 enriched gene ontologies across all three groups (Fig 3C). Specifically, GO enrichment analysis of the 61 overlapping DEGs further highlighted significant involvement in processes related to cell development and membrane components (S1 Table), suggesting the existence of a shared regulatory mechanism that influences cellular structure and function, potentially affecting vesicle dynamics in response to radiation.

### 2.3 Identification of key regulators of exosome biogenesis in irradiated macrophages

**Fig 4:**
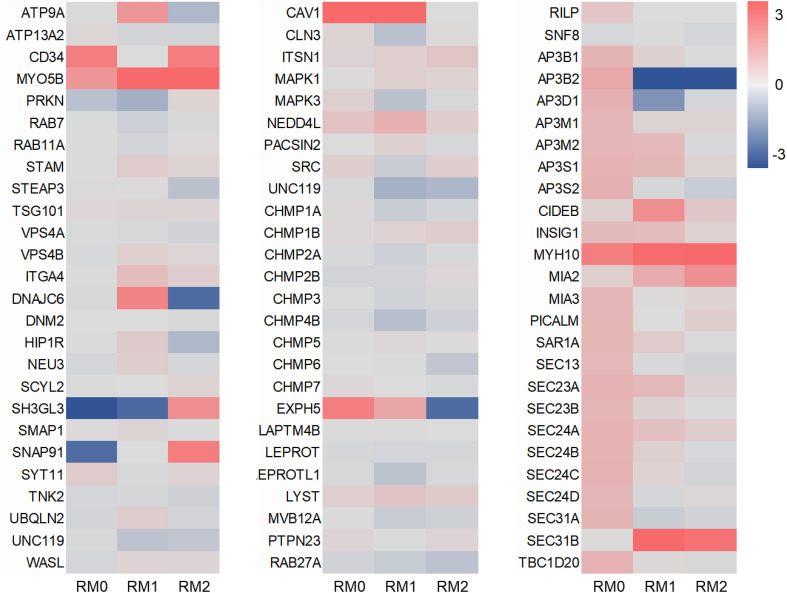
Relative gene expression in irradiated macrophages. Heatmaps showing the expression of genes related to exosome biogenesis. The levels of expression of each gene (log2 fragments per kilobase of transcript per million mapped reads [FPKM]) are indicated by the red/blue scale bar, where red represents up-regulation and blue represents down-regulation.

To further investigate the specific genes involved in radiation-induced exosome secretion, we performed a targeted analysis of the identified DEGs. The 61 overlapping DEGs were filtered by cross-referencing them with the list of exosome biogenesis-related genes obtained from Mouse Genome Informatics (MGI). Through this analysis, we identified eight DEGs whose expression was significantly altered by gamma-irradiation and directly related to exosome release or biogenesis (Fig 4).

**Fig 5:**
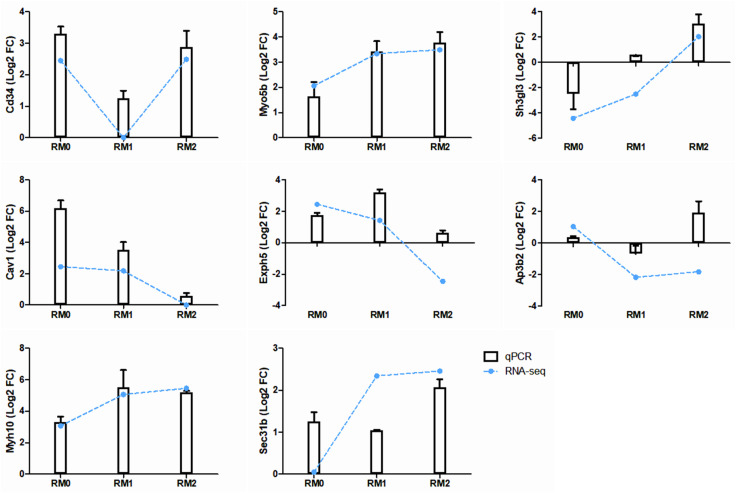
Quantitative PCR validation of genes identified through RNA sequencing. RT-qPCR was used to validate the expression levels of selected genes identified using RNA sequencing (RNA-seq), including Cd34, Myo5b, Sh3gl3, Cav1, Exph5, Ap3b2, Myh10, and Sec31b, in gamma-irradiated RAW264.7 cells (10 Gy, n = 4). Relative gene expression levels are presented as log2 fold change (Log2 FC) values, comparing irradiated macrophages to non-irradiated controls. RNA-seq data are represented by blue dots for comparison with qPCR results.

To validate the RNA-seq data, the expression levels of these eight candidate genes were analyzed using quantitative reverse transcription-PCR (RT-qPCR). As shown in Fig 5, the expression patterns for *Cd34, Myo5b, Sh3gl3, Cav1,* and *Myh10* showed strong accordance between RNA-seq and RT-qPCR results. In contrast, the expression levels of *Exph5, Ap3b2,* and *Sec31* did not consistently correlate. Of particular significance, only Myo5b and Myh10 consistently demonstrated upregulation across all M0, M1, and M2 macrophage subtypes following gamma irradiation. These findings strongly suggest that Myo5b and Myh10 are key candidate genes potentially mediating the enhanced exosome release observed in response to ionizing radiation.

### 2.4. The motor proteins Myh10 and Myo5b regulate exosome secretion in irradiated macrophages

**Fig 6:**
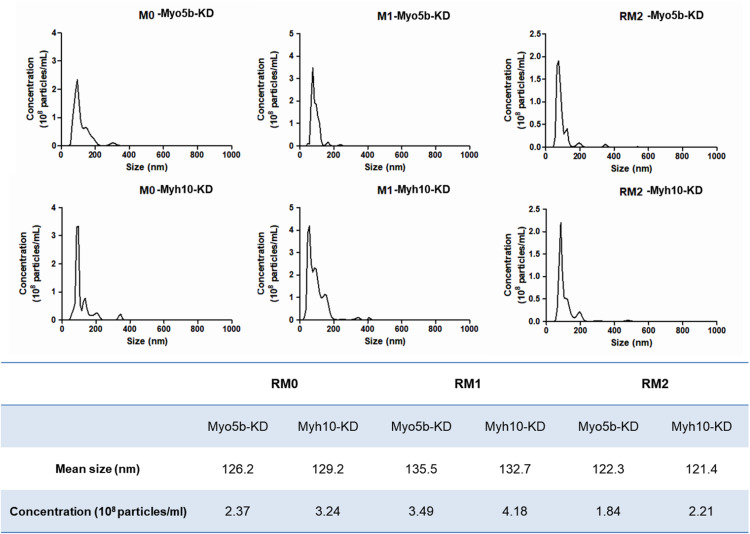
Particle size distribution and concentration of exosomes from macrophages with target gene knockdown. Macrophages with knockdown of Myo5b and Myh10 genes were subjected to 10 Gy gamma radiation. Following 24 h of incubation, exosomes were isolated and analyzed for their size distribution and concentration using the Nanosight 300 system.

To investigate the role of Myo5b and Myh10 in radiation-induced exosome secretion, we performed loss-of-function experiments using siRNAs targeting *Myo5b* and *Myh10*. RAW264.7 cells were transfected with siRNAs, and then cells were subsequently differentiated into M1 and M2 macrophages and irradiated with 10 Gy of gamma radiation. As shown in Fig 6, the knockdown of Myo5b and Myh10 expression levels significantly inhibited exosome release upon gamma irradiation, confirming their direct involvement in the process. In particular, Myo5b knockdown demonstrated high efficacy in regulating exosome secretion. These findings indicated that both Myo5b and Myh10 genes are crucial motor proteins that mediate the exosome release stimulated by gamma irradiation.

**Fig 7:**
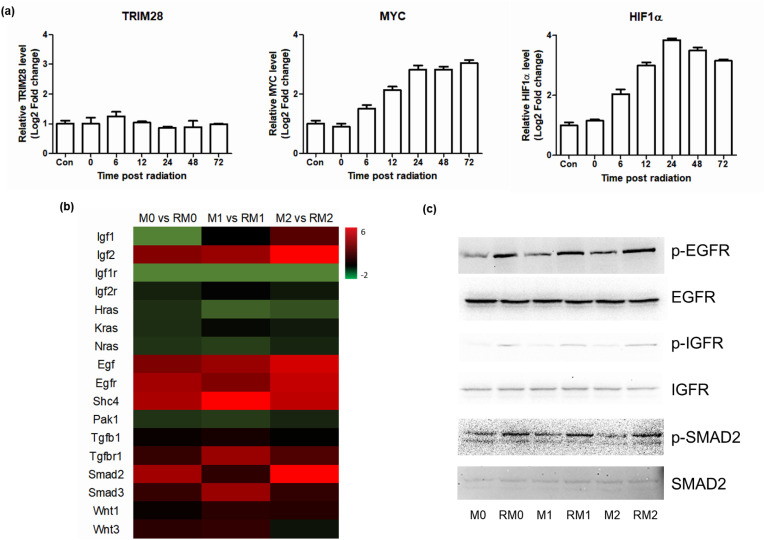
MYC-mediated pathway analysis by RT-qPCR and western blotting. (a) mRNA expression levels (log2 fold change) were assessed in the irradiated group compared to the control group (M0 macrophages). The log2 fold change in mRNA expression levels for TRIM28, MYC, and HIF-1α, which were involved in exosome biogenesis in macrophages after exposure to 10 Gy gamma radiation. **(b)** Heat map of DEGs related to MYC-mediated pathways between irradiated and non-irradiated cells. The scale bar indicates log2 fold change. **(c)** The role of HIF-1α in exosome biogenesis in macrophages after 10 Gy gamma radiation. Western blotting analysis was performed to evaluate the levels of EGFR, p-EGFR, IGFR, p-IGFR, SMAD2, and p-SMAD2 in macrophages treated with irradiated exosomes.

Furthermore, to explore the upstream regulatory mechanisms, we identified potential transcriptional regulators of Myo5b and Myh10 (Fig 7a). This result highlights the complex interplay of these factors in regulating the transcriptional response to radiation. While MYC proto-oncogene (MYC) is well-known for its role in cellular proliferation and hypoxia-inducible factor 1 alpha (HIF1α) in hypoxia response, the identification of MYC in this study suggests that it directly contributes to modulating cellular stress pathways, particularly Myo5b and Myh10 expression, that affect vesicle trafficking and secretion under radiation-induced conditions.

Activation of these receptors appeared to stimulate the MYC pathway, subsequently promoting the expression of Myo5b and Myh10, and thus, enhancing exosome production. This finding suggests a pathway wherein gamma irradiation activates epidermal growth factor receptor (EGFR) and insulin-like growth factor receptor (IGFR), which subsequently upregulates MYC, leading to increased motor protein expression levels, and ultimately, facilitating exosome release.

RNA-seq data also revealed that gamma irradiation consistently increased the expression levels of EGFR and IGFR (Fig 7b), both of which are known to activate downstream signaling pathways. This transcriptional upregulation was further validated by western blotting, which showed a significant increase in the protein levels of EGFR and IGFR in the irradiated groups (Fig 7c). Collectively, these results suggest a compelling pathway in which gamma irradiation activates EGFR and IGFR, which in turn stimulate the MYC signaling pathway. This subsequent upregulation of MYC then promotes the expression of Myo5b and Myh10, ultimately resulting in the observed increase in exosome production and secretion. This proposed EGFR/IGFR-MYC-Myo5b/Myh10 axis represents a novel mechanistic link between radiation exposure and increased exosome release in macrophages.

## 3. Discussion

In this study, Myo5b and Myh10 were identified as key regulators of exosome release from macrophages following radiation exposure. Exosomes are naturally occurring, small vesicles with low immunogenicity, high biocompatibility, and minor toxicity. Additionally, their lipid bilayer structure enables them to function as natural nanocarriers, and their cargo can be modified by engineering their recipient cells. These unique properties render exosomes attractive candidates for diagnostic and therapeutic applications. However, research on the use of exosomes as therapeutic agents is largely limited to preclinical stages. Two major challenges impede the development of exosome therapeutics. First, the difficulty in maintaining consistency and high purity of isolated exosomes hinders drug development. Second, preparing sufficient amounts of exosomes for therapeutic applications remains challenging. Therefore, innovative technologies are essential for effectively improving exosome release and isolation, thereby facilitating the development of exosomes as therapeutic agents. This study confirmed that the overexpression of the motor proteins Myh10 and Myo5b, induced by gamma radiation, significantly accelerated exosome release in polarized and naïve macrophages. Following the stimulation of M1 and M2 macrophages, the cells were exposed to 10 Gy of gamma irradiation. After 24 h of incubation, exosomes were isolated and their morphology and size were examined using electron microscopy and dynamic light scattering (DLS, Zeta-PSA, ELSZneo, Otsuka Electronics, Osaka, Japan). We selected 10 Gy of gamma-irradiation because our previous research indicated that this dose enhances exosome secretion in RAW264.7 macrophages without leading to cytotoxic effects. Exosomes were collected 24 hours post-irradiation, a standard time point that allows for adequate accumulation to facilitate quantitative analysis. While all groups exhibited exosomes with an ideal spherical or cup-shaped morphology, exosomes derived from radiation-exposed macrophages had a diameter of approximately 10–20 nm larger than that of pure exosomes. Furthermore, the NTA results indicated that the concentration of exosomes approximately doubled after exposure to gamma radiation, suggesting that gamma radiation not only influences the biogenesis and secretion of exosomes but also impacts their physicochemical properties. Future studies will investigate various radiation doses and time points to better understand the dose-dependent and temporal dynamics of radiation-induced exosome secretion.

To investigate the molecular mechanisms underlying these observations, RNA-seq was performed to analyze the expression of genes related to exosome secretion. Comparative analysis revealed that 61 differentially expressed genes were consistently upregulated by gamma radiation across different macrophage subtypes. GO enrichment comparisons of the three groups showed that biological processes were associated with differentiation, membrane and vesicle formation, and cytokine-mediated signaling pathways. These findings suggest that gamma radiation induces specific molecular changes in macrophages, particularly in pathways related to exosome secretion. The upregulation of genes involved in differentiation, membrane and vesicle formation, and cytokine-mediated signaling pathways highlights the potential role of these processes in the cellular response to radiation. Further studies are needed to elucidate how these biological processes contribute to the functional changes observed in irradiated macrophages. GO analysis of the overlapping 61 genes revealed similar results (S1 Table). This study provides evidence that gamma irradiation induces transcriptomic changes related to exosome secretion. Furthermore, when comparing the expression levels of genes associated with exosome release and biogenesis, eight genes were upregulated after gamma radiation exposure.

To validate the RNA-seq results, the expression levels of eight selected genes were quantified using qPCR. Overall, there was good correspondence between the RNA-seq and qPCR results. In particular, *Myo5b* and *Myh10* were significantly upregulated after gamma radiation compared to their levels in non-irradiated cells. *Myo5b* encodes a motor protein known as myosin Vb, which plays a crucial role in intracellular trafficking and vesicle transport. It is involved in maintaining the structure and function of various cellular compartments, including the Golgi apparatus and endosomes [24,25]. *MYH10* encodes a non-muscle myosin heavy chain IIB protein that is involved in various cellular processes, such as cell migration and adhesion and cytokine production. It belongs to the myosin II family and functions as a molecular motor that generates force and moves along actin filaments [26,27]. Therefore, these findings suggest that Myo5b and Myh10 are critical contributors to enhanced exosome release, not only in naïve macrophages, but also consistently across both M1 and M2 macrophage subtypes following gamma irradiation.

Further analysis identified MYC and HIF1α as transcription factors that likely regulate Myo5b and Myh10 expression in response to gamma radiation. Notably, MYC plays a role in extracellular vesicle biogenesis, including that of exosomes [28]. In this study, gamma irradiation was found to activate the EGFR and IGFR signaling pathways, which are known to modulate cellular stress responses and growth. Activation of the EGFR/IGFR pathway leads to MYC upregulation, which promotes the expression of Myo5b and Myh10, thereby enhancing exosome release. This elucidated EGFR/IGFR-MYC-Myo5b/Myh10 axis represents a novel signaling pathway that mechanistically links increased exosome secretion and gamma radiation in macrophages. Furthermore, the biological implications of exosome secretion induced by gamma irradiation may go beyond just an increase in vesicle production. Our earlier research showed that exosomes derived from gamma-irradiated macrophages possess improved immunomodulatory and anti-inflammatory properties. This finding supports the potential use of exosomes in immunomodulation and the treatment of inflammatory diseases. Recent studies have shown that γ-irradiation leads to DNA damage and cellular senescence. It is important to investigate whether exosomes released after irradiation are linked to senescence and to assess their potential therapeutic roles during the DNA repair process.

**Fig 8:**
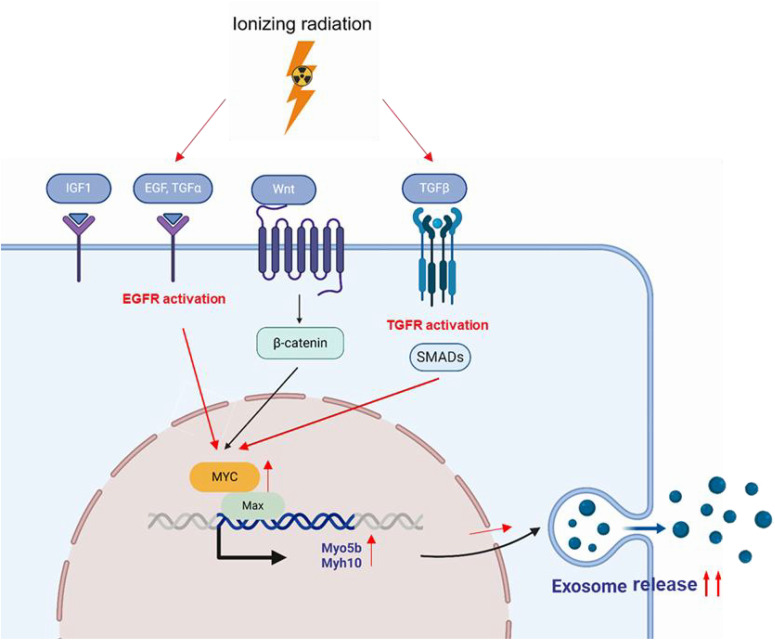
Schematic diagram of EGFR and IGFR activation by ionizing radiation to enhance exosome secretion.

In conclusion, the present study sheds light on the complex mechanisms underlying exosome secretion by macrophages in response to gamma irradiation. By identifying Myo5b and Myh10 as key mediators and elucidating the upstream EGFR/IGFR-MYC signaling axis, our findings provide a novel strategy for overcoming key barriers to exosome therapeutics, particularly those related to yield and production efficiency (Fig 8). Notably, the observed increase in secretion in response to gamma radiation offers a promising approach for addressing the long-standing challenges in the development of exosome-based therapies. Although this study was conducted solely using the murine RAW264.7 macrophage cell line, the physiological relevance of the findings may be limited. Therefore, future studies utilizing in vivo models are crucial to validate the applicability of the results. Additionally, further studies need to include comprehensive proteomic and transcriptomic analyses of radiation-induced exosomes to identify molecular changes and clarify how these alterations may contribute to therapeutic efficacy. Further studies will also incorporate positive controls using known enhancers of exosome secretion to validate and strengthen our experimental results. Our study, therefore, provides foundational insights and a practical methodology for the development of innovative strategies aimed at enhancing exosome secretion, thereby significantly advancing the potential therapeutic applications of exosomes as novel biological agents.

## 4 Materials and methods

### 4.1 Cell culture

Murine RAW264.7 macrophages (Korean Cell Line Bank, Seoul, Korea) were grown in Dulbecco’s modified Eagle’s medium supplemented with 10% fetal bovine serum and 1% (v/v) penicillin/streptomycin (Gibco, Waltham, MA, USA) in a humidified 5% CO_2_ incubator at 37 °C.

### 4.2 Macrophage polarization and γ-irradiation

Macrophages were treated with LPS (100 ng/mL) and IFN-γ (20 ng/mL) for 24 h to stimulate M1 macrophage polarization. To induce M2 polarization, macrophages were treated with IL-4 (20 ng/mL) for 24 h. Polarized macrophages were then irradiated with a dose of 10 Gy using a Gammacell 40 exactor of a 137Cs source at the Advanced Radiation Technology Institute, Korea Atomic Energy Research Institute (Jeongeup, Korea). After irradiation, the macrophages were incubated at 37 °C for 24 h for further studies.

### 4.3 Preparation of exosomes

Exosomes were isolated from conditioned cell culture supernatant using ultracentrifugation method. For the ultracentrifugation approach, the collected culture media underwent sequential centrifugation steps: first at 300 × g for 10 minutes to remove intact cells, followed by 2,000 × g for 20 minutes to eliminate cell debris. The supernatant was then filtered using a 0.22 μm membrane and subjected to ultracentrifugation at 100,000 × g for 70 minutes at 4°C. The resulting exosome pellet was washed with PBS, followed by a second ultracentrifugation under the same conditions before resuspending in an appropriate buffer.

### 4.4 Characterization of exosomes

The hydrodynamic particle size of the exosomes was measured using DLS. The particle numbers were identified using nanoparticle tracking analysis NTA. Quantification of protein in the exosomes was performed using a bicinchoninic acid (BCA) protein assay kit (Thermo Fisher Scientific, Waltham, MA, USA) according to the manufacturer’s protocol. After the exosomes were lysed with an equal volume of radioimmunoprecipitation assay (RIPA) buffer, the lysates were incubated with the BCA reagent for 30 min, and the absorbance was measured at 562 nm. Exosome morphology and structure were visualized using electron microscopy (EM). The exosomes were fixed and negatively stained using 2% uranyl acetate for 1 minute. Sections were mounted on electron microscope grids and examined using a JEOL JEM-1010 electron microscope operated at 120 kV.

### 4.5 Western blotting analysis

Western blotting was performed to determine the expression levels of the exosome marker Alix, the M1 macrophage marker iNOS, the M2 macrophage marker Arg-1, EGFR, IGFR, SMAD2/3, and GAPDH.

Western blotting was performed to determine the expression levels of the exosome markers Alix and CD63, the M1 macrophage marker iNOS, the M2 macrophage marker Arg-1, EGFR, IGFR, p-SMAD2/3, and GAPDH. Briefly, cells were lysed using RIPA lysis buffer (Rockland, Gilbertsville, USA) and total protein was quantified using a BCA protein assay kit. Proteins were separated using 10% sodium dodecyl sulfate-polyacrylamide gel electrophoresis and transferred onto polyvinylidene fluoride membranes. After blocking with 5% skim milk, the membranes were incubated with primary antibodies at 4°C overnight. The membranes were then washed thrice and incubated with secondary antibodies (Cell Signaling Technology, Danvers, MA, USA; 1:2,000) at room temperature for 2 h. Finally, the intensity of the protein bands was visualized using electrochemiluminescence reagents.

### 4.6 Total RNA isolation and RNA sequencing

Total RNA was extracted from polarized and irradiated macrophages using WelPrep™ Total RNA Isolation Reagent (Welgene, Gyeongsan, Korea) according to the manufacturer’s protocol. RNA samples from five randomly selected wells were pooled. The quality and quantity of the purified RNAs were measured using a NanoDrop Lite spectrophotometer (Thermo Fisher Scientific) at an absorbance of 260/280 nm. Quality control samples were sequenced on the HTsat platform. HT-seq counts were used for read counts, and edgeR was used to identify DEGs between samples. GO and annotation analyses were performed using DAVID software.

### 4.7 Quantitative reverse transcription-polymerase chain reaction validation of genes

Following cDNA synthesis using a PrimeScript™ II 1st Strand cDNA Synthesis Kit (Takara, China), RT-qPCR was performed to measure the expression levels of target genes using SYBR PCR Master Mix (Bio-Rad, Hercules, CA, USA), according to the manufacturer’s protocol. β-actin served as the reference gene. All samples were analyzed in triplicate in a single step. Transcript levels were quantified relative to β-actin transcript levels using the 2^− ΔΔCt^ method.

### 4.8 Transfection of small interfering RNAs targeting *Myh10* and *Myo5b*

Small interfering (si) RNAs targeting *Myo5b* and *Myh10* were purchased from Bioneer (Daejeon, Korea). RAW264.7 cells were transfected with 100 nmol/L siRNAs using the transfection reagent Lipofectamine™ 3000 and Opti-MEM™ (Sigma Aldrich, St Louis, MO, USA) according to the manufacturer’s instructions. The cell lysates and supernatants were collected 72 h post-transfection for subsequent experimental measurements.

### 4.9 Statistical analysis

Data are expressed as the mean ± standard deviation. Differences between two groups were assessed for significance using Student’s t-tests, while differences among three or more groups were assessed using one-way analysis of variance. Differences were considered statistically significant at *P* < 0.05. Bar graphs were constructed using GraphPad Prism software (version 6.0; GraphPad Software, San Diego, CA, USA).

## Supporting information

S1 Table GO enrichment analysis of 61 overlapping DEGs. (TIF)

S1 Fig Characterization of polarized macrophages. (TIF)

S2 Fig Full blot images of all western blots reported in Figure 1c and 7c. (TIF)
