# Supplementary figures and images for: Ionizing radiation leads to exosome secretion in macrophages through MYC-mediated pathways
Source: PLoS One. 2025 Nov 5;20(11):e0336322. doi: 10.1371/journal.pone.0336322 (PMC12588456; doi:10.1371/journal.pone.0336322)

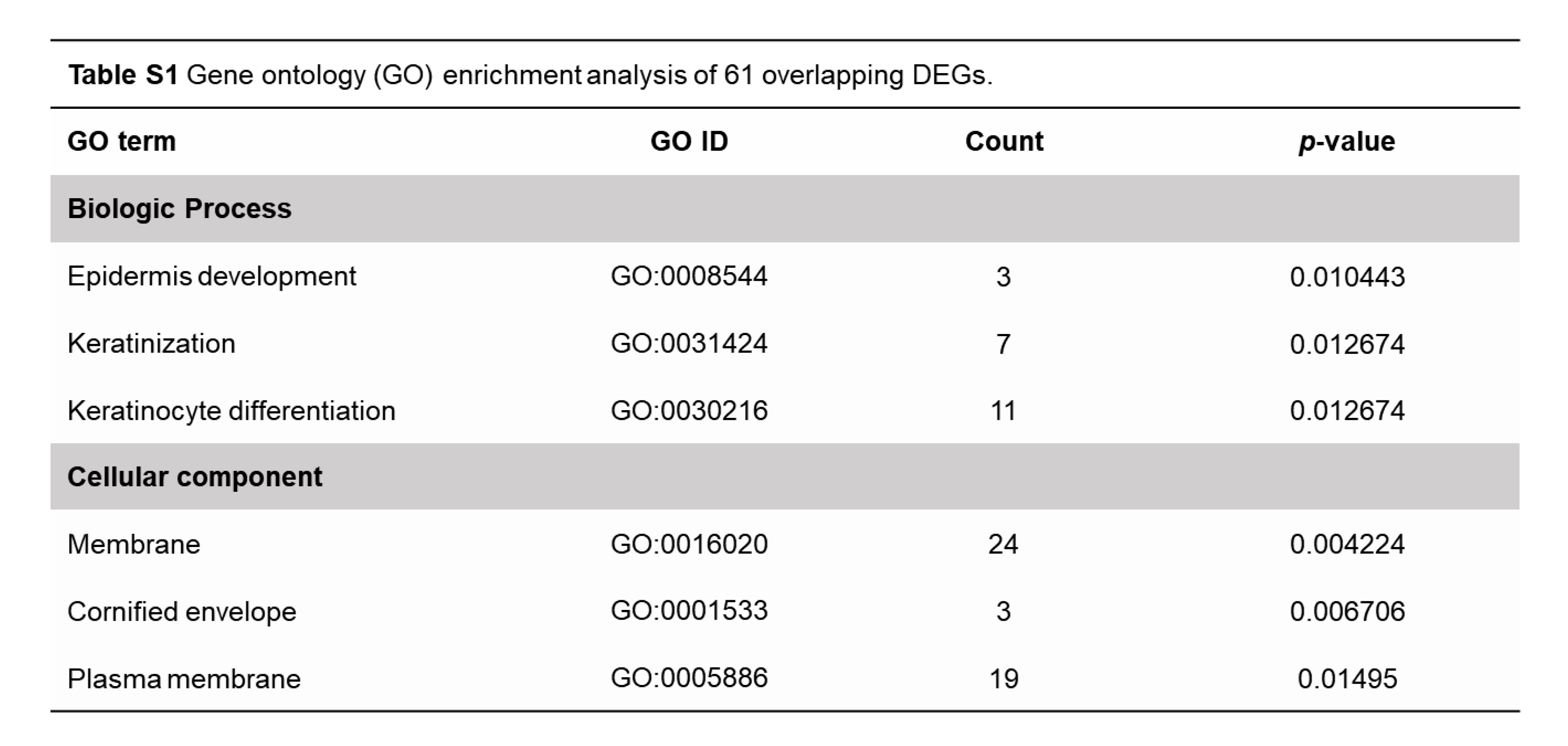

Supplement: S1 Table — (TIF) [file pone.0336322.s001.tif]

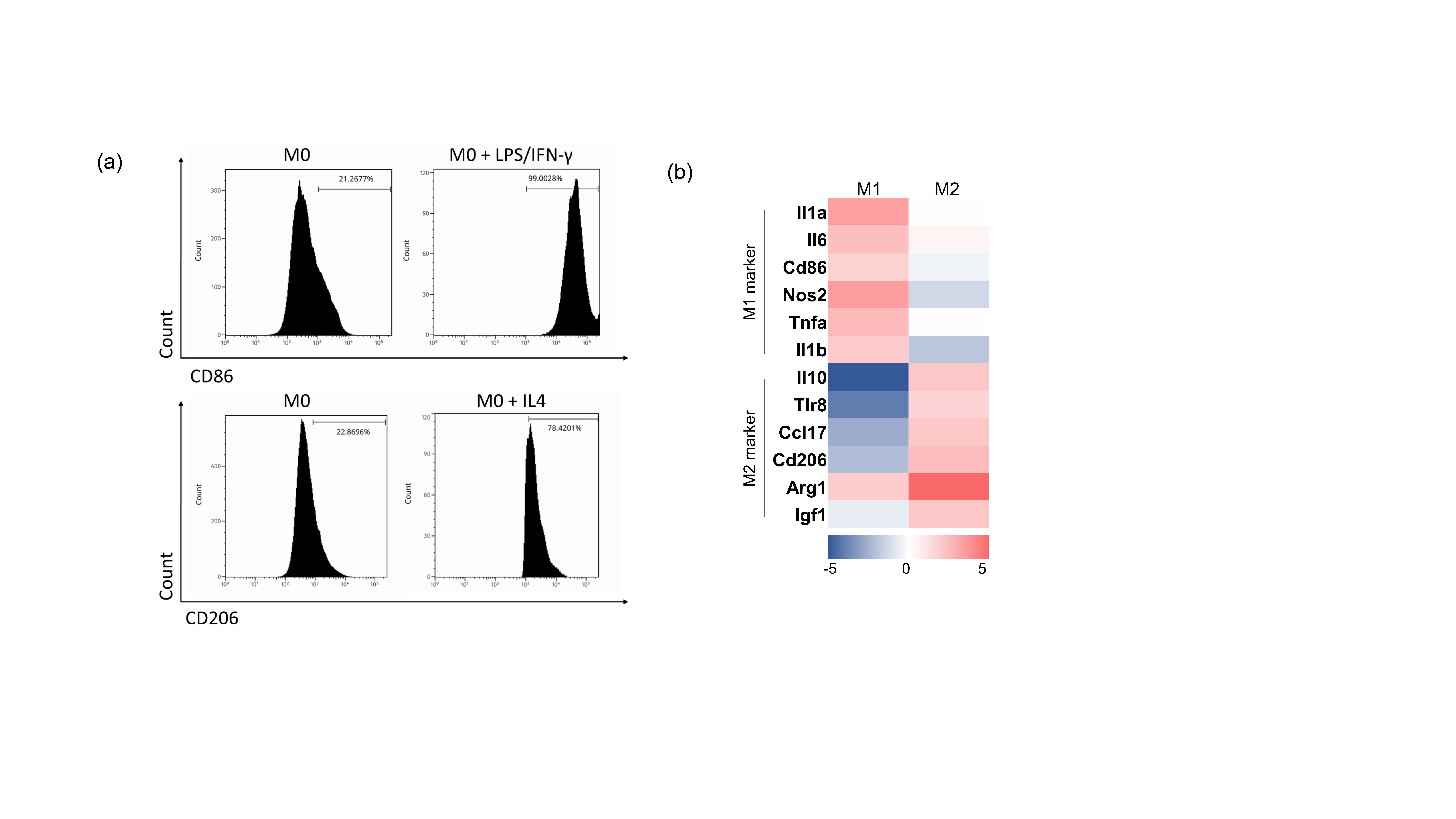

Supplement: S1 Fig — (TIF) [file pone.0336322.s002.tif]

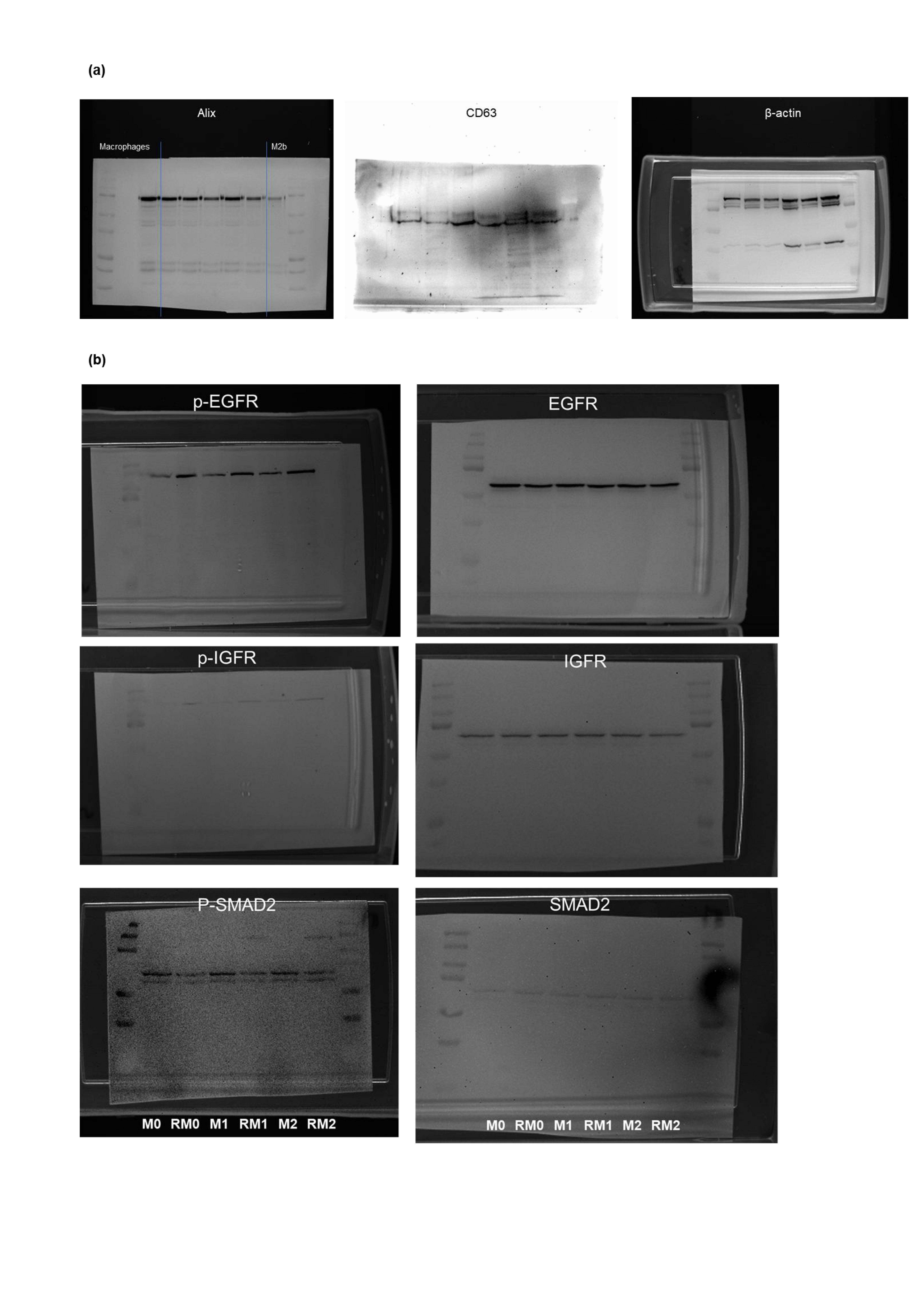

Supplement: S2 Fig — (TIF) [file pone.0336322.s003.tif]
